# A Qualitative Study to Improve How We Partner With Patients and Families in Healthcare Improvement Collaboratives

**DOI:** 10.1111/hex.70312

**Published:** 2025-06-02

**Authors:** Cassandra B. Iroz, Marjorie M. Godfrey, Coua Early, Brad Dell, Abigail Boyle, Erin Tallarico, Julie E. Reed, Julie K. Johnson

**Affiliations:** ^1^ Northwestern University Feinberg School of Medicine Chicago Illinois USA; ^2^ The Microsystem Academy, Lebanon, New Hampshire, USA; School of Health and Welfare Jönköping University Jönköping Sweden; ^3^ The Microsystem Academy Lebanon New Hampshire USA; ^4^ Cystic Fibrosis Patient Honolulu Hawaii USA; ^5^ Cystic Fibrosis Foundation Bethesda Maryland USA; ^6^ Julie Reed Consultancy Ltd; School of Health and Welfare Halmstad University Halmstad Sweden; ^7^ University of North Carolina at Chapel Hill, Chapel Hill, North Carolina USA; Northwestern University Feinberg School of Medicine Chicago Illinois USA

**Keywords:** co‐production, patient engagement, quality improvement

## Abstract

**Background:**

Engaging patients and family members in healthcare quality improvement (QI) is essential to meet the needs of those who receive care. The objective of this study was to describe the experience of patient/family partners (PFPs) in a national QI collaborative and to develop recommendations for best practices for patient engagement.

**Methods:**

We conducted focus groups with PFPs in a national QI collaborative focused on improving transitions of care between cystic fibrosis (CF) and lung transplant programmes. Audio recordings were transcribed verbatim, coded inductively and analysed through thematic analysis. Member checking with PFPs, clinicians and team coaches was used to refine the findings and develop recommendations.

**Results:**

Five PFPs participated in two focus groups, which revealed that PFPs (1) were motivated to participate as members of the QI team because they felt deeply connected to their CF care teams and wanted to help other patients, (2) felt engaged in the QI collaborative and appreciated the sense of community, support from the team coach and the opportunity to take ownership of projects, and (3) suggested improvements related to timing of meetings, compensation, being mindful when discussing sensitive information and setting clear expectations. Member checking revealed the need for equitable recruitment processes and tailoring the role to the individual participants. The findings were used to develop and change processes in the collaborative.

**Conclusions:**

The structure of the national QI collaborative supported patient/family partnership through structured meetings and a focus on building relationships of mutual respect. The findings demonstrated the need for a more equitable recruitment process, better expectation setting and customisation of the role to the individual skills, needs and preferences of the participants.

**Patient or Public Contribution:**

Patients and family members of people living with CF participated in this study through focus groups and member checking. One CF patient (B.D.) is a co‐author of this paper and contributed to data analysis, sensemaking, writing and editing.

## 1 Background

Engaging patients and families in healthcare quality improvement (QI) is essential to meet the needs of those receiving care. Patient engagement can occur on a continuum from consultation to shared leadership [1] and has led to innovative outcomes for redesigning guidelines, systems and practices [2, 3]. Many patient/family partners (PFPs) have reported positive experiences, learning about the healthcare system, affirming their right to advocate and feeling like meaningful members of the team [4, 5]. However, there are challenges to PFP engagement, including uncertainty about best practices, discomfort in speaking up and lack of time, training and funding [2, 3, 6]. Despite attempts to engage PFPs, patients sometimes have little input and power in decision‐making [7]. Even as patient engagement has become more common, there is still much to understand about the complexities of how to increase the likelihood of a positive, mutually beneficial experience.

The cystic fibrosis (CF) community has a rich history of patient engagement. PFPs have contributed to setting priorities [8, 9], developing research [10, 11, 12, 13, 14, 15], creating guidelines [16, 17, 18, 19, 20, 21] and QI [22]. Founded by parents of children with CF, the Cystic Fibrosis Foundation (CFF) has consistently prioritised the needs of patients and families [23]. CFF facilitates the Partnership Enhancement Program (PEP), a training programme on relationship‐centred communication [24], and Community Voice, where individuals sign up to share their perspectives through various activities, including surveys, focus groups and committees [25, 26]. In 2017, findings from Community Voice highlighted the need to improve transitions of care for people with CF referred for lung transplant [20]. To address this, clinical guidelines and a national QI collaborative were developed, engaging PFPs to ensure the changes were responsive to the needs of the community [27, 28].

Within this context of a national QI collaborative developed in response to the needs of the community and supported by an organisation founded by parents of children with CF, we explored the experiences of PFPs who participate in QI teams. While patient engagement is a central value of the CFF and the QI collaborative, we wanted to understand the actions taken by local teams, the structures that support or inhibit patient engagement, and the opportunities for improvement, from the perspective of PFPs. To identify challenges and opportunities in engaging PFPs in QI work and to develop recommendations for best practices, we conducted focus groups with PFPs and gathered community feedback through member checking.

## 2 Methods

### 2.1 Study Design

We conducted a qualitative study to understand the experience of PFPs in a national QI collaborative. The study is reported following the Consolidated Criteria for Reporting Qualitative Research (COREQ) guidelines [29] and was approved by the University System of New Hampshire Institutional Review Board (UNH‐IRB‐FY2021‐60).

### 2.2 Study Setting

The Cystic Fibrosis Lung Transplant Transition Regional Dissemination Network (CF LTT RDN) was a QI collaborative across the United States and Canada aimed at improving processes, communication, education and relationships between CF and lung transplant teams [28]. Through the CF LTT RDN, 10 transplant centres worked with 43 referring CF centres. Multidisciplinary QI teams, consisting of physicians, nurses, social workers, physical and occupational therapists, respiratory therapists, nutritionists and pharmacists, worked with team coaches [30] to apply microsystem theory [31]. They attended regular local, regional and national meetings to share QI experiences and lessons learned. Each QI team was expected to include at least one PFP and independently decided how to engage them. There was no standard PFP recruitment or orientation process.

### 2.3 Participants and Sampling

We used a convenience sampling approach [32] by sending emails to all PFPs in the CF LTT RDN (including 24 patients and 3 family members), inviting them to a focus group to share their experiences and provide feedback. Participants provided electronic written consent before the focus group.

### 2.4 Data Collection

We conducted two virtual focus groups in March 2023. The focus groups were facilitated by J.J., a senior health services researcher with expertise in qualitative methods, and C.I., a doctoral candidate in healthcare quality and patient safety with training in qualitative methods. Both J.J. and C.I. participated as team coaches in the CF LTT RDN, but neither had an established relationship with any of the focus group participants. Focus groups lasted approximately 60 min, were semi‐structured and included prompts about successes, challenges, what kept PFPs engaged and how to sustain PFP involvement (Appendix A). The focus groups were audio recorded and transcribed verbatim.

### 2.5 Data Analysis

Building on the interactive research model [33], team coaches, leaders and PFPs assumed hybrid practitioner–researcher roles, providing information about their experiences in the CF LTT RDN and contributing to the analysis and sensemaking of the findings. We inductively developed a codebook, based on research questions and in‐depth review of the focus group transcripts (Appendix B). C.I. independently coded each transcript, using MAXQDA 2022 (VERBI Software, 2021) to facilitate data management. Themes and sub‐themes were identified through iterative rounds of discussion with C.I., J.J., J.R. and M.G., integrating the insights from the focus groups and their firsthand experience within the collaborative. Preliminary themes were then reviewed by the rest of the authorship team (C.E., B.D., A.B. and E.T.) to refine findings.

After analysing the focus group data, we conducted member checking by presenting the findings to people with CF, multidisciplinary teams and team coaches at the CF LTT RDN National Summit in June 2023. We also presented the results through a poster at a prominent national meeting, the North American Cystic Fibrosis Conference (NACFC) in November 2023, where we received feedback from other researchers, multidisciplinary healthcare professionals and family members of people with CF. These comments were used to refine our findings and develop resources and recommendations.

## 3 Results

### 3.1 Themes From the Focus Groups

Five PFPs in the CF LTT RDN attended the focus groups. From these focus groups, we identified three overarching themes: motivation to participate in QI, successes that PFPs experienced in the CF LTT RDN and challenges and opportunities for improvement (Table 1).

**Table 1:** Themes and sub‐themes.

Themes	Sub‐themes
Motivation to participate	Desire to help other CF patients
	Connection to their care teams
	Have seen positive changes
Successes and practices to continue	Feel heard and valued
	Collaborative environment
	Ability to take ownership over projects
	Team coaches and the structure of the meetings
	Strong sense of community
Challenges and opportunities for improvement	Timing of meetings
	Lack of compensation
	Difficultly of discussing sensitive information
	Limited expectation setting, preparation and training
	Some patients preferred to attend only when their input was necessary

### 3.2 Theme 1: Motivation to Participate

PFPs were motivated to participate as members of the QI team because they felt deeply connected to their CF care teams and wanted to help improve care for other patients. We identified three sub‐themes related to motivation.

First, they participated because of a *desire to help other CF patients*. The participants described a strong sense of community with others living with CF. Some talked about a lifelong commitment to engagement in the CF community. One participant shared that their mother had been active in the CF community and they were continuing that tradition in the CF LTT RDN. The participants talked about peer support and advocacy work they do in addition to their roles in the CF LTT RDN.

‘I think it's my sense of community and wanting to give back.’

Patient 1

Second, they felt *connected to their care teams* with whom they had long‐term relationships. The PFPs talked about the deep relationships they built with their CF care teams including relationships that spanned years or decades working with the same physicians, nurses and multidisciplinary team members. They described these relationships as ones of trust and understanding.

‘They have saved my life. So, it's a different working relationship. But it was nice to help them take apart a process that I've lived through and maybe provide some value to it.’

Patient 3

Third, they were motivated to stay with the programme because they had *seen positive changes*. They expressed seeing improvements because of the work of the QI team, which motivated them to continue their efforts.

‘Hearing that, so far, it has had some positive changes for patients themselves definitely keeps me sticking with it’

Patient 5

### 3.3 Theme 2: Successes and Practices to Continue

PFPs reported feeling engaged with the CF LTT RDN and appreciated the sense of community, support from the team coach and the opportunity to take ownership of QI projects. We identified five sub‐themes of success.

First, the teams asked for PFPs' opinions, making them *feel heard and valued*. Each participant talked about how they appreciated when they were directly asked for their perspective.

‘I think they enjoy some of my ideas and we get to get information out to the patients and that kind of thing. So, I think they appreciate that.’

Patient 2

Second, the teams fostered a *collaborative environment*. Using applied microsystem theory, QI teams were trained to conduct meetings with a lens of multidisciplinary collaboration rather than hierarchy. Rotating roles, each member was encouraged to take turns leading and participating. PFPs reported feeling like they were part of the team, not peripheral observers.

‘I think my team nailed it. I think they were great with working with us as patients.’

Patient 1

Third, patients could *take ownership over projects* they were passionate about. These projects allowed PFPs to leverage their skillsets to contribute to the QI team. For example, one focus group participant described their experience leading a project to create a tool guiding patients through the transplant referral process.

‘One of the things that we took on … was a patient's life perspective [for the] website and guide to expectations of the whole process.’

Patient 3

Fourth, they enjoyed working with *team coaches and the structure of the meetings*. Team coaches met with each team regularly and guided them through improvement projects. Each team received training in effective meeting skills, which included having timed agendas, rotating meeting roles (e.g., leader, facilitator, recorder and timekeeper) and collaboratively setting ground rules. PFPs reported appreciating these structures. They also reported that the team coach acted as an advocate for the patient voice and would prompt the PFPs to share their perspectives.

‘I like having an outside coach that's not part of either team because they can ask questions from a different perspective.’

Patient 4

Fifth, during the Covid‐19 pandemic, some teams continued to stay connected and built a *strong sense of community*. Early in the pandemic, clinical teams were stretched thin as many of them were reassigned to work in the intensive care units. In some cases, QI meetings became less frequent, but teams continued to stay in touch. Some participants reported that the meetings were less focused on moving through the steps of QI and were instead used as a time to connect and support one another. Demonstrating the power of patient engagement, one PFP spearheaded the creation of a heartfelt video message from patients to their healthcare team, expressing gratitude and offering encouragement.

‘It was more of kind of a sense of community that I think actually really ended up helping us grow as a team … it was checking in with each other on a human level, not necessarily with work.’

Patient 1

### 3.4 Theme 3: Challenges and Opportunities for Improvement

PFPs made suggestions for improvements related to the timing of meetings, compensation, discussions of sensitive information and expectation setting. We identified five sub‐themes related to challenges and opportunities for improvement.

First, the *timing of meetings*, which were generally during the workday, made it difficult to participate. While this was not an issue for some participants, others reported that it was difficult to attend meetings while working. They also stated that their availability changed along with their health (e.g., while on disability, they had more flexibility with their schedule, but after returning to work, they were less often able to attend).

‘The time that they schedule [the meeting] for is convenient for them, but I don't know if they ever considered how it would be convenient for the patient.’

Patient 4

Second, participation as a PFP took a significant amount of time, which was *uncompensated*. None of the focus group participants reported receiving any financial compensation for their roles as PFPs. Some participants voiced frustration, feeling that there was an unequal balance as the healthcare professionals were doing the QI work as part of their paid employment, and the PFPs were contributing equally without compensation.

‘The doctors and physical therapists and whoever, they get paid for it. It's their job. But what about us?’

Patient 5

Third, they wanted the team to be *sensitive* to information that might be difficult for patients to hear (e.g., poor outcomes or death). One participant described feeling very upset after attending a CF LTT RDN meeting where a presenter shared a story of losing their spouse. The participants also discussed how the clinical team members can speak about outcomes such as mortality and transplant failures pragmatically with a degree of professional detachment. However, for a person living with or awaiting a transplant, these stories and statistics could be difficult to hear. The focus group participants stated that they wanted to know ahead of time what topics were going to be discussed so they could choose whether to participate.

‘It's hard to hear certain things about CF or hear it repeatedly … like a husband whose wife was no longer with us … maybe [it is best] to split up those groups whenever possible when discussing really difficult sensitive issues.’

Patient 1

Fourth, there was a need for improved *expectation setting, preparation and training*. Most of the focus group participants reported that they did not have a good understanding of their role and the structure of the CF LTT RDN when they agreed to participate.

‘When I joined…. I didn't know it was going to be a weekly thing. I didn't know it was going to be a long‐time thing.’

Patient 4

Regarding training, some participants wanted resources that were easy to understand so they could learn QI terminology and more actively participate.

‘For somebody who doesn't really do quality improvement, a Quality Improvement for Dummies [would be helpful].’

Patient 4

However, others stated they were not interested in learning QI tools and that they preferred only to attend meetings and give their input.

Fifth, some PFPs did not want to attend every meeting and *preferred to attend only when their input was necessary*. Some described their ideal approach as one where PFPs give input on priorities, the team works on the details of QI and then comes back to the PFP for their feedback. Some described not wanting to attend meetings focused on internal processes or interprofessional communication. This was particularly relevant as their capacity to attend meetings changed along with their health status. One participant described using the meeting agendas to decide when they would attend.

‘Just maybe not having us participate at such a granular level but calling us in when bigger level concepts should be shared.’

Patient 1

## 4 Member Checking

After analysing the focus group transcripts, we received feedback from various stakeholders. At NACFC (2023), family members of people living with CF illuminated the issue of ‘cherry picking’ PFPs. They believed that the clinical teams made assumptions about who would be a good PFP, but did not consider others who would be interested. This topic was further explored through discussions with the team coaches, which highlighted how there was no standard process for PFP recruitment. The current practice of selecting individuals to invite also brought up concerns about diversity, equity and inclusion of those chosen to participate. The overarching suggestion from these discussions was that there should be a more inclusive process for recruiting PFPs, perhaps where individuals apply to join the team. At the CF LTT RDN National Summit (2023), some multidisciplinary team members shared challenges at their institutions for engaging PFPs, including the need for volunteer training, paperwork and other administrative policy barriers (including those related to compensation).

Sharing the results of the focus groups with additional PFPs highlighted the need to customise the PFP role. While most of the focus group participants liked the ideas of less frequent involvement, during the member checking process, other PFPs stated that they enjoyed being engaged in all aspects of the QI work, regardless of whether the tasks were patient‐facing or internal to the clinical teams. During the focus group, one participant talked about how they were familiar with QI tools from their professional career and were able to participate in more of the technical aspects of improvement. During the member checking process, team coaches shared stories of other patients who had advanced skills in Microsoft Excel and developed a tracking tool. Other PFPs organised and presented at sessions at the CF LTT RDN National Summits. However, others preferred to only attend meetings. This diversity of experiences highlights the need for flexibility for PFPs to participate in ways that align with their wants, needs, abilities and availability.

Additional discussions with the CF LTT RDN community clarified challenges related to compensation and expectation setting. While there was widespread agreement on the importance of fairly compensating PFPs for their contributions, participants also recognised the complexity of this issue. For some PFPs, receiving financial compensation could jeopardise their eligibility for disability benefits. This highlights the need for careful consideration and flexible compensation models to ensure inclusivity and avoid unintended consequences.

## 5 Response to Findings

Given what we learned from the focus groups and member checking, we developed resources to guide QI teams in patient engagement. We first collected resources from the Cystic Fibrosis Learning Network (CFLN) [22], Cystic Fibrosis Success with Therapies Research Consortium [34] and Cystic Fibrosis BreatheCon Planning Committee [35]. From these tools and our research findings, we created the Patient and Family Partner Engagement Guide, which includes seven key documents (Table 2; Appendix C). We also compiled resources for PFPs, including Microsystems at a Glance [36], Electronic QI Modules, Improvement Workbooks, Quality by Design [31] and other resources available at clinicalmicrosystem.org. In response to the concerns about compensation, the CFF is developing a policy and accompanying guidelines to help CFF staff implement the PFP compensation policy in a consistent way.

**Table 2:** Patient and Family Partner Engagement Guide.

Item	Contents
Process Flow	Outlines the process for PFP engagement Includes reviewing the discussion checklist Provides a recommendation to offer a buddy
What is a Patient Family Partner?	Description of what is a PFP Successful invitation process History of the PFP recruitment process
Checklist For Engaging Patient and Family Partners	Includes developing a plan, meeting with individuals who are interested, completing an expectations worksheet, identifying a buddy, asking the PFP for feedback, reviewing available resources and planning time for regular check‐ins
Patient and Family Partner Recruitment Email Template	A template of an email to send to all patients in the practice to inform them of the PFP role
Patient and Family Partner ‘Internal’ QI Team Information Form Template	Description and purpose of the QI team Time commitment, meeting dates and meeting format Expectations of PFPs Compensation
Patient and Family Partner Onboarding Guide	Advice for the first meeting with PFPs Introductions and getting to know one another Clarify expectations and answer questions
Patient and Family Partner Role Description (For Patient/Family Partner)	What to expect from the QI meetings Expected contributions Estimated time commitment

*Note:* The full Patient and Family Partner Engagement Guide is available in Appendix C and at clinicalmicrosystem.org.

**Figure 1:**
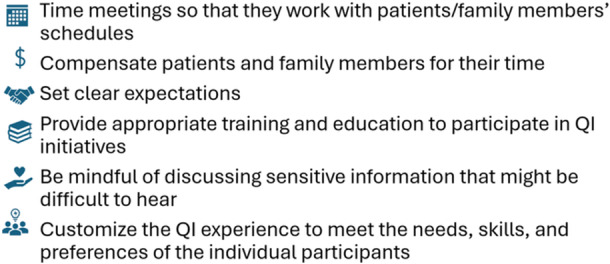
Recommendations for engaging patient/family partners in quality improvement.

Based on the findings, we propose six best practices for QI teams looking to engage PFPs (Figure 1).

## 6 Discussion

Listening to the voices of PFPs, we learned that they were motivated to contribute to QI teams because they felt deeply connected to their care teams and wanted to help others, felt appreciated by their teams and supported by the team coach, and experienced challenges regarding the timing of meetings, lack of compensation, hearing difficult information and gaps in expectation setting. Conversations with additional PFPs, clinicians and team coaches revealed the importance of developing equitable recruitment processes that honour the diverse expertise in the community and the need to customise the QI experience to the skills, capacity and preferences of the individual participants.

Many of our findings align with what others have reported about the benefits and challenges of engaging PFPs in healthcare improvement. Similar to Godfrey et al., we found that patients and caregivers want to know what is expected of them, including time commitment [37]. Others have also reported challenges with the timing of QI meetings [5, 38]. Based on their experience engaging PFPs in QI, the CFLN has created best practices, including establishing clinician readiness, facilitating an inclusive PFP recruitment process, supporting multiple PFPs on each team, providing QI training, selecting projects that reflect PFP priorities, facilitating access to information, encouraging an inclusive and safe involvement, compensation, and disseminating work in open‐access venues [39]. Our focus group study identified many of the same best practices, in addition to the PFP perspective that it can be difficult to participate in discussions of sensitive information and that it is important to tailor the role to the individual. These findings contribute to the growing literature on the importance of including the beneficiaries of care in QI interventions to enhance the relevance and outcomes of QI interventions [40].

A key finding from our study is the critical importance of personalising the QI experience for each PFP. This means recognising and accommodating their individual preferences, needs and abilities to create meaningful and valuable engagement. By prioritising this personalised approach, we can empower PFPs to contribute effectively and derive greater satisfaction from their involvement in QI efforts. This flexibility is also reflected in the CFF Community Voice programme, which offers opportunities ranging from membership on committees to responding to surveys and providing feedback on draft materials [16, 25, 41]. To optimise collaboration, teams should carefully determine the most appropriate level of engagement, considering the specific needs of the project, clinical team and PFP [1, 7, 42, 43, 44]. In our study, we found that the team coach played a crucial role in helping teams work with PFPs in a way that was individualised and mutually beneficial.

Our research highlights a critical need for a more inclusive PFP recruitment process. Participants expressed the importance of seeking out and welcoming diverse perspectives and ensuring a broad range of voices are included. Furthermore, the current structure of QI, with meetings held during traditional work hours and a lack of compensation for PFPs, creates significant barriers to inclusivity. This can inadvertently exclude those who could benefit most from QI, particularly individuals with caregiving responsibilities or limited flexibility, or those facing financial constraints [43]. Beyond inequities in who participates, there is an inherent power imbalance in patients being invited by the clinicians who hold the power, which has led to criticisms of exclusivity and tokenism [45, 46]. There have been calls to move away from traditional models that narrowly focus on feedback to models that empower PFPs through equal and reciprocal relationships [43]. The CFF has recommended avoiding tokenism by ensuring there is more than one PFP on the team, engaging them throughout the duration of the project and creating a team environment that builds trust [41].

The participants in this study discussed issues of compensation for PFPs, which is a common issue across healthcare. Other PFPs have described the importance of compensation in addressing equity and power imbalances, acknowledging differences in motivation, respecting vulnerability, demonstrating commitment and removing barriers to engagement [47]. Recommendations exist for financial and non‐financial compensation [47, 48, 49]. Researchers and QI leaders searching for best practices on PFP compensation can look to a recent review of guidance documents [48]. All the reviewed documents recommended offering non‐financial compensation (e.g., training opportunities) and 95% recommended offering financial compensation, including honoraria, gift cards, salary and stipends, with the recommended amount ranging from $12 to $50 per hour.

While many of our findings echo what others have reported about the benefits and challenges of engaging PFPs in QI, we also found some key differences. The structure of the CF LTT RDN, use of effective meeting skills and support from the team coach likely mitigated some of the challenges reported by others such as unorganised meetings, little follow‐up, lack of understanding of the healthcare organisation, frustration with the slow decision‐making process, availability of sufficient resources and communication skills of the QI team [5, 38]. The collaborative environment likely supported prerequisites for positive PFP involvement, including a focus on relationships and communication, training in QI principles, shared power and leadership, non‐hierarchical, multidisciplinary collaboration, and mutual recognition and respect [6, 50].

## 7 Limitations

These findings should be interpreted considering several limitations. First, only five PFPs participated in the focus groups. PFPs who were less engaged or had negative experiences might have been less likely to respond to the request, thus leading to positivity bias. Second, PFPs were selected by their healthcare teams and might not represent the diverse experience of those living with CF. Third, a common limitation of focus groups is the tendency for socially acceptable opinions to emerge and for dissenting opinions to be withheld [51]. To mitigate this limitation, the trained moderators actively sought input from all participants and created space for dissenting opinions. Fourth, the involvement of CF LTT RDN team coaches and leaders in the interactive research model may have biased the interpretation of the results, given their vested interest in the success of the programme. However, the hybrid practitioner‐research members did not shy away from critiques (e.g., the perception of unfair compensation), suggesting a genuine commitment to learning and improvement [33].

## 8 Future Directions

Future research is needed to explore the broader experience of patient/family involvement in QI and test the recommendations we proposed. First, given our limited sample, additional work could explore a broader and more diverse sample, perhaps capturing the experiences of patients and family members who are less often engaged in QI and research. Second, mixed‐methods evaluations could be conducted to assess interventions based on the recommendations of this study, including (1) more equitable recruitment processes, (2) compensation models for PFPs and (3) implementation and effectiveness of the included Patient and Family Partner Engagement Guide.

## 9 Conclusion

The structure of the national QI collaborative, the use of effective meeting skills and the support of a team coach facilitated an environment of trust and mutual respect that leveraged the strengths of PFPs. Even within the context of a QI collaborative created by the direct influence of patients and family members, there is still a need to attend to ongoing processes and practices and embed these processes into local QI teams. PFPs highlighted the need to improve recruitment, compensation and resources available to QI teams and PFPs. While at times it might feel time‐consuming, messy or intimidating to include patients and families as true partners in improvement efforts, from our experience, the rewards far outweigh the challenges.

## Author Contributions

**Cassandra B. Iroz:** conceptualisation, methodology, data curation, formal analysis, investigation, writing – original draft, writing – review and editing, validation, project administration, visualisation. **Marjorie M. Godfrey:** conceptualisation, methodology, investigation, validation, funding acquisition, writing – original draft, writing – review and editing, project administration, formal analysis, supervision, resources, data curation. **Coua Early:** conceptualisation, data curation, investigation, writing – review and editing, validation, project administration, resources. **Brad Dell:** writing – review and editing, validation, investigation, resources. **Abigail Boyle:** writing – review and editing, validation, resources, funding acquisition. **Erin Tallarico:** writing – review and editing, validation, resources, funding acquisition. **Julie E. Reed:** writing – review and editing, conceptualisation, methodology, data curation, formal analysis, validation, investigation, resources. **Julie K. Johnson:** supervision, writing – review and editing, writing – original draft, visualisation, validation, methodology, conceptualisation, investigation, formal analysis, data curation, resources.

## Ethics Statement

This study was approved by the University System of New Hampshire Institutional Review Board (UNH‐IRB‐FY2021‐60).

## Consent

All participants provided electronic written consent.

## Conflicts of Interest

Two of the co‐authors (E.T. and A.B.) are employees at CFF, and the rest of the co‐authors have received compensation from CFF for their roles as researchers in the CFF LTT RDN. None of the authors has any additional conflicts of interest relevant to the submitted article.

## Supporting information

PtFamPartner AppendixA ModeratorGuide.

PtFamPartner AppendixB codebook.

PtFamPartner AppendixC PFPEngagementGuide.

## Data Availability

Data is available upon request due to privacy/ethical restrictions.
