# Supplementary material for: A Qualitative Study to Improve How We Partner With Patients and Families in Healthcare Improvement Collaboratives
Source: Health Expect. 2025 Jun 2;28(3):e70312. doi: 10.1111/hex.70312 (PMC12128466; doi:10.1111/hex.70312)
Supplement: Supplementary file 1 — PtFamPartner AppendixA ModeratorGuide. [file HEX-28-e70312-s003.docx]

**CF LTT RDN “Experience and Sustainability” Virtual Patient and Family Focus Groups**

**March 2023**

Groups that did not attend the RDN National Summit in June 2022 in Pittsburgh, Pennsylvania will be contacted to provide additional information and perspectives.

1. Patients and families who have participated in the CF LTT RDN will be invited to participate in a virtual focus group.

**The aim of the semi-structured interviews and the virtual focus group is:** to understand interviewee views on participating in the RDN and the relationships, partnerships, and networks that have formed, achievement, successes, challenges, and sustaining improvement in the RDN.

**Inclusion and Exclusion Criteria:**

Our study sample includes patients and family members who have participated in the CF LTT RDN program from 1-4.5 years. Participants eligible for the interviews include English-speaking adults of any gender.

1. All CF LTT RDN listed patients and family members of the CF LTT RDN will receive an email invitation to participate in a virtual focus group interview via Zoom. Participants will complete a Qualtrics registration form if they voluntarily agreed to participate. See attached for listing.

**Consent:**

An email to confirm participant registration will be sent out before the scheduled virtual focus groups to provide virtual focus group date, time and Zoom link. All groups will receive a request to complete the consent form in advance of the focus group.

*Good Afternoon:*

*Thank you for signing up for a virtual CF LTT RDN Focus Group. The aim of the focus group is: to understand participant views on participating in the RDN and the relationships, partnerships and networks that have formed, achievement, successes, challenges and sustaining improvement in the RDN.*

*Your Virtual Focus Group will take place on date. Please see connection information below.*

*In order to participate in this research study, we will need to receive your consent. Please click here to review and sign the consent form by date.*

*Please do not hesitate to reach out with any questions or clarifications that you have.*

*We look forward to seeing you!*

**Financial Compensation:**

There will be no special financial compensation to participate in the focus groups.

**Potential Benefits to Participants:**

There will be no direct benefit to participants who agree to participate. Participants who participate in the focus groups will benefit the program as a whole, which benefits future CF LTT RDN activities and could potentially benefit them by improving their insights and interventions.

**Risks to Participants:**

Participation includes sharing one’s beliefs, opinions, and feelings regarding the CF LTT RDN program. Therefore, this study is of no more than minimal risk to participants. The foreseeable risks and inconveniences include giving of their time (which should be minimal) and potentially emotional or psychological if they have had previously developed negative associations with CF LTT RDN participation.

Participation in the focus groups will be voluntary and in no way linked to their professional role or participation in the CF LTT RDN. They will be informed of this prior to them agreeing to participate, and they will be allowed to drop out of the study at any point without consequences. If participants withdraw from the research, a research team member will ask if any data collected up until that point may be used in the research.

**Data Monitoring Plan to Ensure the Safety of Participants:**

This study is of no more than minimal risk to subjects and data will be safeguarded as detailed above. Our study team has access to numerous data sources with sensitive information and a long-standing history of using appropriate safeguards to assure data is kept safe and secure. Our systems are regularly tested, and individuals are required to keep up to date with federal and state regulations regarding the use and management of protected health information. All members of the research team have completed Human Subject Protection certification.

***Interview Guide for Patients and families who have participated in the CF LTT RDN will be invited to participate in a virtual focus group.***

**Session PREP:** (Recording by phone will use Phone App: Voice Memo or if Zoom recording during session)

- Before each focus group, the interviewer will check to confirm if online consent was completed. **If online consent wasn’t completed, the interviewer will do a verbal consent.**

There are no right or wrong answers, so please feel free to speak freely. Also, we assure you that we will not connect names or sites to any quotes or information we use when we report the analysis.

Participation in this focus group is voluntary and you may decide not to answer questions and if you wish, you may leave the focus group at any time.

We would like to record the focus group because that makes it easier for us to pay attention to on the conversation instead of taking notes. We plan to have the recordings transcribed and any identifiers removed so that we can conduct a thematic analysis of the focus groups. We assure you that you can talk freely. Is it okay with you if we record this session? Phone recordings will be uploaded and deleted. Digital backup recording will be uploaded and deleted.

All interviews will be audio-recorded if the participants are amenable. The audio recorder may be turned off at the participant’s request at any point during the interview. If a participant does not wish to be recorded, the interview will proceed without any audio record. Any incidentally obtained identifiable data will be removed so that the data will be de-identified and kept on a password protected drive on a secure network.

Current CF LTT RDN teams, leaders and coaches were invited to share their experiences at the National Summit in Pittsburgh, PA in June 2022 and semi-structured interviews are being conducted with teams who declined to continue in the RDN, are/were on the fence about continuing participation in the CF LTT RDN or are waiting to continue the RDN at a later date.

Do you have any questions for us before we start?

Before we turn on the recorders, please introduce yourself and tell us which team you are a part of.

1. Notify participants that you will now start recording the session.

START RECORDING ON 2 DEVICES (personal telephone app**/Voice Memos-iPhone** /or Zoom recording)

1. Describe format of the session so participants know what to expect: We will be together for around 75 minutes to discuss in more depth your reflections and role that relationships, partnerships and networks have played in the success, sustainability and challenges with the RDN collaborative.

**Discussion Guide:**

We will NOT collect any protected health information on providers during these interviews. The discussion guide outlined below includes the type of questions that will be asked, and the topics explored together as a group. Exact wording may vary during the sessions. The important component is that participants have the opportunity to convey what is important to them about their experience of partnership working with the RDN collaborative. As such, the focus groups may not cover all questions and may include additional questions other than those listed below that is in line with the direction of the group’s discussion.

Please have each member respond to number one (#1) around the circle:

1. To start, let’s hear about each of your experiences as a patient/family partner in the RDN.
   1. Which site do you work with?
   2. How long have you been a patient/family partner?
   3. [clarify if they are a patient or a family member-should be on sign up]
   4. [if patient], have you received a lung transplant?
2. Could you describe a **successful situation** where you felt everyone worked together well and you felt a sense of achievement in working together?
   1. What was going on?
   2. What made this successful?
   3. What left you with a sense of achievement?
3. What **keeps you engaged** and working with your partners?
   1. What motivates you to continue to contribute your time to this work?
4. We know that it can be difficult to sustain this improvement work overtime and that many patient or family partners have left the program at various times for various reasons. What ideas do you have on how we can **improve sustainability** of the RDN and keep patients and families engaged on the improvement teams?
   1. What do you think are the greatest challenges to sustainability of the improvement team?
   2. Have you received any **compensation** for your role as a patient/family partner?
      1. Do you think compensation would make a difference in people’s decision to participate?
   3. Did you receive any **training** to be a patient/family partner?
      1. What kinds of training, if any, do you think would best prepare people to participate as patient/family partners?
5. Trust is often cited as an important component of inter-collaborative working. What does trust look like within the RDN collaborative?
   1. How would you say trust has been developed?
   2. How has trust been sustained over time? Throughout the pandemic?
6. Have you encountered any **challenges** in working with your team? If so, how did you resolve those?
7. Now that you’ve had a chance to reflect about the RDN and sustaining relationships and improvement over time, what **stands out for you as being most important**?
8. Thinking of an **ideal world**, what would being a patient/family partner look like? What changes do you think we can make to the program to ensure that patients and family member can contribute to the fullest extent?

**CONCLUSION:**

Is there anything else that you would like to tell us about, or make sure that we learn about while we are here today?

Thanks for agreeing to take time to meet with us today.

**CLEANING AND TRANSCRIBING DATA:**

Focus group transcripts will be scrutinized to remove all identifiers and an iterative analysis using QDMAX will be conducted. When there are differences, consensus discussions will take place between the researchers.
