# Supplementary material for: A Qualitative Study to Improve How We Partner With Patients and Families in Healthcare Improvement Collaboratives
Source: Health Expect. 2025 Jun 2;28(3):e70312. doi: 10.1111/hex.70312 (PMC12128466; doi:10.1111/hex.70312)
Supplement: Supplementary file 3 — PtFamPartner AppendixC PFPEngagementGuide. [file HEX-28-e70312-s002.docx]

**Patient and Family Partner Engagement Guide**

**Version Date: May 16, 2025**

**Also available on clinicialmicrosystem.org**

**Contents**

**Documents for Quality Improvement (QI) Teams**

Process Flow Page 2

What is a Patient Family Partner? Page 3

Checklist for Engaging Patient and Family Partners (PFP) Page 4

Patient and Family Partner Recruitment Email Template Page 6

Patient and Family Partner “Internal” QI Team

Information Form Template Page 7

Patient and Family Partner Onboarding Guide Page 9

**Document for Patient and Family Partners**

Patient and Family Partner Role Description Page 10

**QI Team**

*Patient and Family Partner (PFP) Engagement*

*Process Flow*

Discuss and confirm with QI Team, People with ACFLD and Family members their interests to contribute, flexibility, and commit to meeting PFPs where they are on their terms.

Reach out to the practice’s patients/families, i.e. direct invite, posted notices in waiting area, etc.

Review PFP discussion checklist with the team

(see pages 4-5)

Review number of interested PFP, their availability and determine number to accept. Decide which applicants to invite or not. If not invited, option to discuss other methods of involvement

Offer a “buddy” (a member of the QI team) connection for those who express interest to learn more

Onboarding with team and “buddy”

**QI Team**

*What is a Patient and Family Partner?*

A Patient and Family Partner (PFP) is a person, at least 18 years of age, with cystic fibrosis (CF) or a family member of a person with CF invited by their CF or Transplant Center to partner with the center’s quality improvement (QI) team in the Cystic Fibrosis Lung Transplant Transition Regional Dissemination Network (CF LTT RDN) and CF LTT RDN Expansion programs. The PFP will enhance the improvement by providing a unique perspective about CF LTT care, processes, and improvements based on personal experiences.

**What is the Patient and Family Partner Recruitment Process?**

The PFP Recruitment Process is a process for identifying a person with CF, or a family member of a person with CF who is interested in partnering with the CF or Transplant Center’s CF LTT RDN QI team in the role of a Patient and Family Partner. The goal is to include PFPs from a diverse variety of backgrounds and experiences.

**A successful PFP Invitation process includes:**

Describing and sharing the PFP role and impact to CF care and services with all patients/families to invite their consideration as a PFP. Consider posting an announcement on a listserv, newsletter and a poster in the practice along with sharing an example of QI activities and how the PFPs were involved, i.e. review new educational materials etc.

Sharing PFP documents including a QR Code for interested PFP members to express interest to participate.

- - Offer an opportunity for PFP who expresses interest to connect and talk with other PFP members (peer connection) before meeting with the CF or Transplant programs to further understand the role and expectations. (See Checklist for Engaging Patient and Family Partners for more detailed information on providing a QI Team member “buddy” and expectations.)

**History of the Development of the Patient and Family Partner Recruitment Process**

Historically, most CF and Transplant Centers have recruited patient and family members through a CF or Transplant QI team-initiated selection process. CF and/or Transplant program providers would identify patient/family members they thought might be interested in the role, and with whom the QI team might partner with.

Although the team-initiated selection process has successfully recruited patient and family members over time, concerns have arisen that the process may not have been equitable and have not identified all potential candidates who may be interested in the role of the Patient and Family Partner.

Patient and Family Partners have shared that sometimes they feel obligated to agree to participate as a PFP out of gratitude for their team's care, so our aim is to ensure all potential PFPs have an opportunity to explore the role, express interest, and participate in the selection process out of their own interests without pressure.

**QI Team**

*Checklist for Engaging Patient and Family Partners (PFPs)*

*This is a preliminary checklist of topics to discuss with the team and patient and family partners. Once an invitation is extended to the program patients and families, those who express interest would arrange time to meet in person or virtually, to clarify, discuss and co-design their partnership and involvement with the improvement team based on their interests, time, and availability.*

**Items to consider:**

1. Develop a plan to invite practice patient and family partners (PFP) including clear expectations of being a PFP.
2. Take the time to consider the families that are cared for and what they bring to the program. The inclusion should emphasize a variety of races, ethnicities, incomes levels, sexual orientation, socio-cultural aspects, etc.
3. Schedule a time to meet with those who express interest to discuss and co-design their partnership and involvement
   1. Discuss how they want to be involved, e.g., CF improvement meetings, learning sessions, regional and national meetings.
   2. Identify interests and skills the PFP would like to contribute.
   3. Assure the PFP that there is flexibility in participating with the improvement team depending on their situation and ability.
   4. Discuss with the PFP the frequency of joining the improvement meetings e.g., weekly, every other week, monthly. PFPs do not need to attend all improvement meetings and by communicating what is convenient for them will help the improvement team focus on specific PFP interests at the meetings they can attend.
   5. Discuss days/times that work best for the PFP to participate in the meetings. Emphasize there is flexibility for the improvement team to adjust meeting day/time to meet their needs and availability.
   6. Discuss length of involvement. Several months, 6 months, one year depending on their availability including periodic evaluations and feedback based on their experience as a PFP and their own life situation.
   7. Identify what the most convenient method is for the PFP to join the meeting e.g., in person, Zoom, telephone, etc. and if they have the equipment, infrastructure (internet) to join easily. Important to share the sessions can be recorded if they are unable to join.
   8. Identify communication preferences with the PFP including frequency and type of communication. Do they want to be included all QI Team correspondence or only topics they are interested in?
4. If a patient or family member agrees to participate, discuss mutual expectations of the PFP with the improvement team and leaders. Document the expectations by completing the “Expectation” worksheet while discussing.
5. Identify an improvement team “buddy" who can meet with the new PFP in advance of the first meeting and between meetings, as needed, to support the PFP by answering questions and providing clarifications.
6. Ensure improvement meeting agendas are sent in advance (follow the 7-step meeting process with advance agendas set at the end of each meeting) and include partner interests and topics in the agendas they want to attend - confirm partner interest and comfort with agenda topics.
   1. Be mindful of sensitive information discussed in team meetings (especially around outcomes, life expectancy and mortality) and alert the PFPs if these topics are on the agenda to help them decide if they want to participate or not. Communicate with improvement team members the need to be mindful about these topics.
   2. The PFP is an active member of the Quality Improvement team. Include them in all aspects of the meeting including meeting roles, action items, and other activities that they express an interest in.
7. During the meetings, seek PFP feedback and be receptive to their suggestions and comments. Their unique perspective adds value and important considerations to the quality improvement work.
8. Provide clarification, information and invite the PFP to review the improvement resources e.g., Microsystems at a Glance, Electronic QI modules, RDN website, textbooks, worksheets and other content specific to the improvement collaborative.
9. Identify which team member will respond to PFP emails in a timely manner to build trust and communicate that their time to participate in the work is valued. (Consider the “buddy” in this role).
10. Plan time every other month to review partner participation, what is working and what can be improved and other ideas.
11. Include PFP as interested in national meeting preparations. As applicable, the partner (if a family member) may be able to attend in person depending on location and situation. Encourage virtual attendance if attending in person is not possible or if the PFP is a patient who cannot attend due to infection control practices.
12. Encourage the PFP to communicate with, convene and include other patients and family members.

**QI Team**

*Patient and Family Partner (PFP)*

*Initial Patient/Family Partner (PFP) Recruitment Email*

Recruitment Email Template Sample

(a customizable template to be adapted to the setting)

**Subject Line**: Invitation to work with Your Care Team on Quality Improvement

Dear [Patient/Family],

We are looking to ***invite people with CF and/or family members of a person with CF who might be interested in joining our Quality Improvement (QI) Team as a Patient and Family Partner (PFP)***. PFPs will join their local CF care center or Lung Transplant program to learn together what care delivery currently is and to identify opportunities for improvement based on the PFP experiences.

The PFP role will work locally with our QI team at the care center, and if there is interest and availability, with other care centers in the region, and nationally.

To learn more about the PFP role, expectations, and goals of improvement you can scan this QR Code [teams can create QR codes or links to information about their program].

If you have interest in participating as a Patient/Family Partner, or are curious to learn more, please Scan the QR Code.

[teams can create QR codes or links for potential participants to express interest]

If you have any questions, please email [Contact Info]

Thank you,

[Signature]

**QI Team**

*Patient and Family Partner Talking Points*

*Information Form Template*

**Date**

| **CF or Lung Transplant Improvement Team Name** | [Your Center Name] QI Team for the Cystic Fibrosis Lung Transplant Transition Regional Dissemination Network – Expansion (CF LTT RDN Expansion) |
| --- | --- |
| **Description of Team** | [List the names and roles of your QI Team]  We are looking for additional Patient and Family Partner(s) to join the QI team. |
| **Improvement Team Description/ Purpose** | [List your center’ QI activities]  The role of the Patient and Family Partner is to help their QI team understand what it is like to receive care to identify opportunities for improvement. |
| **Time Commitment varies determined in partnership with the PFP** | - 1-hour weekly, bi-weekly, or monthly QI team meeting - 1-hour monthly RDN Regional Meeting - 1-hour “Special Topics”, 2-4 times per year, as per individual interest - Participation in national meetings, either virtual or in person. The national meeting is approximately 24 hours over three days. PFPs can choose their level of participation. - Virtual (Patient Partner) or In Person participation in national meetings. |
| **Meeting Dates to be explored with the PFP** | QI Team to discuss with PFP to identify frequency, day, and time the PFP can participate. After confirming PFP participation, the QI team can fill in the remaining dates/times. |
| **Expectations of PFP** | - Monthly time commitment approx. 1-2 hours - Actively participate in QI meetings using Effective Meeting Skills - Share health care experiences with team members - Co-produce solutions to better meet needs of all patients - Participate in new improvement initiatives - Connect and collaborate with other Patient/Family Partners in the RDN Network |
| **Meeting format** | - Virtual (phone/video) participation. - Local in-person meeting or National Summit attendance as applicable per infection control guidelines. |
| **Honorarium Information** | [Identify honorarium timeframe (monthly, quarterly, etc.)]  Note: Determine with PFP type of compensation they would prefer e.g., money, gas card, travel or other options taking into consideration individual disability and or tax limitations.  If money, the amount and method is TBD |

**QI Team**

*Patient and Family Partner (PFP) Onboarding Guide*

While inviting Patient and Family Partners, it is important to talk to Partners about expectations of the work and to gauge their involvement interests and abilities.

**Things to Keep in Mind:**

**Consider creating an onboarding video/voice over PowerPoint welcome for the potential PFP to review first, followed by the “buddy” Zoom/phone call.**

- Remember to use the Onboarding Zoom/phone call as a chance for relationship building – spend time getting to know the partner. “Meet them where they are at.”
- Be sure to explore interests and ability appropriately – this can ensure that the opportunity is the right fit based on interests, skills, and availability of the Partner.
- If the role is not the right fit, mention other ways the Partner can get involved – encourage them to visit [www.cff.org/Get-Involved](http://www.cff.org/Get-Involved) or other ways to provide feedback through other PFPs, suggestion boxes, etc.

**Advice for CF/Transplant Program when meeting with potential PFP**

- Thank the Partner for responding to the interest survey for this opportunity to partner to improve care.
- Start with introductions–share a little about yourself and ask about the Patient/Family Partner
- Ask general questions about desired involvement and interests
  - How do you want to get involved or support the CF community?
  - What topics are you interested in?
  - What skillsets do you have?
- Cover the basics and clarify any questions
  - Explain the improvement opportunity and the confirm PFP level of participation and interest.
  - Clarify that the PFP is a representative of the practice’s patient and family population and not necessarily the needs of the specific family.
  - Outline tasks and time commitment agreed upon with the PFP.
  - Orient the PFP to past, current and future improvement themes, and processes to help generate interest.
  - Discuss how the PFP participation and specific feedback will be used to help with the improvement efforts.
- If ready to confirm interest in the PFP, ask if the PFP would be willing to serve in the role
  - If role is accepted, describe next steps and timeline
  - If role is declined, thank the member for interest and remind them of other ways to get involved

**Patient and Family Partner**

*QI Role Description*

This document describes the roles and responsibilities of **Patient and Family Partners** (PFPs) to participate in the CF Lung Transplant Transition Regional Dissemination Network (CF LTT RDN) and CF LTT RDN Expansion Program. PFP(s) are partners of the Quality Improvement (QI) Team.

Although the specific tasks of the PFP(s) may vary, the main role is to partner with the Quality Improvement team to improve care delivery processes. The PFP participates as a QI team member to represent the patient and family perspective in the improvement work.

Key areas of activity for the PFP are as follows:

**PRACTICE QUALITY IMPROVEMENT**

- A “Buddy” from the QI team will partner with the PFP to guide orientation to the Quality Improvement method and resources
- The quality improvement (QI) method also includes using effective meeting skills
- Partner with interprofessional clinical care team members to identify opportunities to improve care processes and interventions to better meet the needs of all people with CF and their families.
- Participate in weekly, biweekly, or monthly QI team meetings and monthly CF LTT RDN Regional meetings. (To be determined by the PFP)
- Participate in network-wide meetings. (To be determined by the PFP)

**CONTRIBUTE PATIENT AND FAMILY LIVED EXPERIENCE AND PERSPECTIVE**

- Offer the patient/family perspective as an equal contribution in all discussions.
  - Provide insights to the team about the care system’s impact.

**ESTIMATED TIME COMMITMENT* can vary depending on PFP ability to participate.**

- 1-hour per weekly, bi-weekly, or monthly local team meeting
- 1-hour monthly network-wide meetings
- Participation in annual national meetings.
